# Differential effects of cow dung and its biochar on *Populus euphratica* soil phosphorus effectiveness, bacterial community diversity and functional genes for phosphorus conversion

**DOI:** 10.3389/fpls.2023.1242469

**Published:** 2023-09-14

**Authors:** Yuxian Fan, Guanghui Lv, Yudong Chen, Yaling Chang, Zhoukang Li

**Affiliations:** ^1^ College of Ecology and Environment, Xinjiang University, Urumqi, China; ^2^ Key Laboratory of Oasis Ecology of Education Ministry, Xinjiang University, Urumqi, China; ^3^ Xinjiang Jinghe Observation and Research Station of Temperate Desert Ecosystem, Ministry of Education, Jinghe, China

**Keywords:** cow dung biochar, P effectiveness, microbial diversity, P functional gene, phosphate-solubilizing bacteria

## Abstract

**Introduction:**

Continuous monoculture leading to soil nutrient depletion may cause a decline in plantation productivity. Cow dung is typically used as a cheap renewable resource to improve soil nutrient status. In this study, our purpose was to compare the effects of different cow dung return methods (direct return and carbonization return) on soil microbial communities and phosphorus availability in the root zone (rhizosphere soil and non-rhizosphere soil) of *P.euphratica* seedlings in forest gardens and to explore possible chemical and microbial mechanisms.

**Methods:**

Field experiments were conducted. Two-year-old *P.euphratica* seedlings were planted in the soil together with 7.5 t hm^-2^ of cow dung and biochar made from the same amount of cow dung.

**Results:**

Our findings indicated that the available phosphorus content in soil subjected to biochar treatment was considerably greater than that directly treated with cow dung, leading to an increase in the phosphorus level of both aboveground and underground components of *P.euphratica* seedlings. The content of Olsen-P in rhizosphere and non-rhizosphere soil increased by 134% and 110%, respectively.This was primarily a result of the direct and indirect impact of biochar on soil characteristics. Biochar increased the biodiversity of rhizosphere and non-rhizosphere soil bacteria compared with the direct return of cow dung. The Shannon diversity index of carbonized cow manure returning to field is 1.11 times and 1.10 times of that of direct cow manure returning to field and control, and the Chao1 diversity index is 1.20 times and 1.15 times of that of direct cow manure returning to field and control.Compared to the direct addition of cow dung, the addition of biochar increased the copy number of the phosphorus functional genes *phoC* and *pqqc* in the rhizosphere soil. In the biochar treatment, the abundance of the phosphate-solubilizing bacteria *Sphingomonas* and *Lactobacillus* was significantly higher than that in the other treatments, it is relative abundance was 4.83% and 2.62%, respectively, which indirectly improved soil phosphorus availability.

**Discussion:**

The results indicated that different cow dung return methods may exert different effects on phosphorus availability in rhizosphere and non-rhizosphere soils via chemical and microbial pathways. These findings indicated that, compared to the direct return of cow dung, biochar return may exert a more significant impact on the availability of phosphorus in both rhizosphere and non-rhizosphere soils, as well as on the growth of *P.euphratica* seedlings and the microbial community.

## 1 Introduction

*Populus euphratica*, one of the world’s oldest rare tree species, is currently classified as endangered. This tree is a valuable forest resource in arid desert areas and plays an extremely important role (Ling et al., 2017). According to Ling et al. (2015), the Tarim River Basin in China’s Xinjiang Uygur Autonomous Region is the most widespread and abundant region worldwide. *P.euphratica* is considered to be the most ancient indigenous tree species in the Xinjiang Desert region. As a constructive and dominant species, it has advantageously adapted to cold, drought, high-temperature, saline alkali, and wind sand resistance (Pan et al., 2016). *P.euphratica* has emerged as a favored species for vegetation restoration and artificial afforestation in dry desert regions, leading to extensive plantation of artificial seedlings in forest nurseries (Xu R. al., 2023; Yu et al., 2023b). However, the harsh nature of its planting habitat, which is characterized by arid climatic conditions and original sandy soil properties, leads to a scarcity of soil nutrient elements and thereby weakens the ability of artificially planted *P.euphratica* seedlings to retain fertilizer, causing these seedlings to deteriorate with time. In addition, continuous planting at the same site depletes soil nutrients, particularly phosphorus, which greatly limits the growth of *P.euphratica* seedlings, thereby restricting the renewal and development of *P.euphratica* populations and hindering ecological restoration projects in arid desert areas (Yu et al., 2023a). Although soil phosphorus deficiencies may be countered via supplementation with phosphate fertilizer, this approach, which involves unusually high economic costs, eutrophication of water bodies, and increased greenhouse gas emissions, is both ecologically destructive and unsustainable (Jin et al., 2023).

Phosphorus is a nutrient that is critical for plant growth and development. Phosphorus promotes the growth of *P.euphratica* seedling roots, increases their absorption capacity, hastens their growth, and improves their stress resistance (He et al., 2023). Plant-usable phosphorus mainly comes from soil, wherein the total content of phosphorus is only 0.02 – 0.2% (Qin et al., 2006), of which over 80% is fixed in soil and cannot be absorbed by plants (Gao et al., 2019). To enhance seedling growth and afforestation, large amounts of phosphorus fertilizers are applied to the soil. A majority of chemical phosphorus fertilizers that are applied to soil remain insoluble, leading to a low phosphorus fertilizer utilization rate, typically ranging from only 10% to 25% (Yu et al., 2021). Phosphate rock is a finite resource, and proven phosphorus reserves and current extraction rates indicate that the mining period in China may last less than 30 years (Cooper et al., 2011). Additionally, global reserves of phosphate rock are projected to be exhausted in approximately 50 – 100 years. Therefore, there is an urgent need to find new materials that can replace some of the phosphate fertilizer used or improve the effectiveness of soil phosphorus (Penuelas et al., 2023).

Biochar, a porous material that is rich in organic carbon, has been widely applied for agricultural and environmental protection purposes in recent years (Kurniawan et al., 2023). It is produced via the pyrolysis (300 – 1000°C) of various biomass sources, including but not limited to crop straw, animal manure, and wood(Qian et al., 2023). They are known for their stability and refractory properties. Biochar improves the availability of phosphorus in soil through different mechanisms(Jin et al., 2023; Xue et al., 2023). During pyrolysis, volatilization of carbon and cleavage of O-P bonds convert organic phosphorus, such as phospholipids, into inorganic phosphorus (phosphates), which can be absorbed and utilized by plants, making biochar a direct source of soil phosphorus (Liu et al., 2019). Typically, negatively charged biochar surfaces are capable of directly binding cations, including Ca^2+^, Al^3+^, and Fe^3+^, which reduce the processes that lead to soil phosphorus adsorption and precipitation (Xu G. et al., 2014; DeLuca et al., 2015a). The activities of Ca^2 +^, Al^3 +^, and Fe^3 +^ depend on soil pH, and biochar indirectly increases the availability of soil phosphorus by changing soil pH (Gao et al., 2019). The unique highly porous structure of biochar confers other advantages, such as improved soil structure and enhanced soil venting and drainage (He et al., 2019). In addition to enhancing the availability of phosphorus, biochar also augments the content and effectiveness of essential elements required by other plants, and enhances soil nutrient activity (Gul and Whalen, 2016; Wang et al., 2018). It also affects soil microorganisms by increasing soil water retention, internal surface area, and enzyme activity, thereby changing soil nutrient availability(Hei et al., 2023; Liu et al., 2023).

Furthermore, biochar promotes the growth and reproduction of soil microorganisms by providing them with a large number of substrates and more diverse habitats, which exert a beneficial effect by increasing the activity of soil microorganisms and altering soil microbial communities (Bolan et al., 2023). Soil microbial community structure undergoes various changes following biochar-related modification (Lehmann et al., 2011; Muhammad et al., 2016; Huang et al., 2017; Gao et al., 2019). Addition of biochar to soil significantly altered the composition of the microbial community, increasing microbial biomass and functional diversity (Rutigliano et al., 2014; Han et al., 2017). Application of biochar greatly increased bacteria in the rhizosphere soil of spring barley and enhanced the efficacy of soil phosphorus by more than 100 times (Fox et al., 2016). However, the structure of the soil microbial community did not change dramatically following biochar addition (Nguyen et al., 2018; Yu et al., 2018). The effects of biochar addition on soil microorganisms also affected soil properties (Gu et al., 2023; Ni et al., 2023; Xu W. et al., 2023).

Phosphate-solubilizing microorganisms are a class of microbes that demonstrate an ability to convert insoluble phosphorus molecules in the soil into soluble phosphorus, which is easily absorbed by plants. The two main categories of phosphate-solubilizing microorganisms in the soil are phosphate-solubilizing fungi, such as *Penicillium* spp., and phosphate - solubilizing bacteria, such as *Bacillus* spp. (Deb et al., 2016). While some phosphate - solubilizing bacteria are able to mineralize organic phosphorus, other phosphate-solubilizing microorganisms can solubilize inorganic phosphorus molecules. Phosphate-solubilizing microorganisms dissolve insoluble inorganic phosphorus through genes, such as *gcd* and *pqq, pqqB*, and *pqqC* (Andreeva et al., 2011), whereas phosphate uptake and transport are mainly carried out through multi - component phosphate transporters encoded by pho genes, such as *phoC* and *phoD* (Liu et al., 2018). The functional genes *phoC, phoD, gcd*, and *pqqC*, which are involved in microbial phosphorus transformation, act as typical molecular markers (Jiang et al., 2023).

Although people are increasingly aware of the potential positive role of biochar in agricultural systems, the impact of biochar on forestry systems is not fully understood (Ge et al., 2023; Zhang et al., 2023). At present, most of the research reports on biochar improvement are about acidic soil (Zhou et al., 2020; Tian et al., 2021; Yang and Lu, 2022), and there are few studies on the effect of biochar on phosphorus availability in alkaline soil (grey desert soil) in arid areas. Based on the planting of *Populus euphratica* seedlings in arid areas, this study studied the effects of different cow dung returning methods on soil phosphorus availability and microbial community in different root zones of *Populus euphratica* seedlings. Although the effects of cow dung and biochar as organic fertilizers and soil amendments, respectively, on soil microbial communities and phosphorus availability have been extensively studied. (Wang et al., 2021; Nahidan and Ghasemzadeh, 2022), few studies have comprehensively compared the effects of different returning methods of cow dung (direct returning and carbonization returning) on phosphorus availability and microbial community in different root zones of *Populus euphratica* seedlings planted in alkaline soil in arid areas. Using cow dung as a pyrolysis feedstock has advantages over plant feedstock, especially in arid regions where plant biomass is scarce. Since grazing cattle is the main land use in drylands, cow manure in such ecosystems is sometimes considered a waste and environmental hazard, but will be turned into a resource. Therefore, assessing the potential impact of biochar extracted from animal manure on the productivity of *populus euphratica* seedlings is worth investigating. In addition, quantitative PCR (qPCR) technique was used to detect and evaluate the abundance of key functional genes (phoC, phoD, gcd, pqqC) in phosphate transforming microorganisms. The microecological mechanism by which biochar changes the soil microbial community structure in the root of *populus euphratica* seedlings to improve the availability of phosphorus was revealed from a more microscopic perspective, and the potential microscopic effects of biochar on the planting of *populus euphratica* seedlings in arid regions were further understood, so as to guide future planting of *populus euphratica* seedlings in arid regions and provide relevant correction information for the application of bovine dung biochar. We hypothesized that (i) cow manure and its corresponding biochar have different effects on the availability and composition of phosphorus in soil. (ii) The improvement of soil phosphorus availability was more significant by returning cow dung to field than by returning cow dung directly to field. (iii) The improvement of soil phosphorus activity by cow manure carbonization may be the effect of biochar on the chemical and microbial processes of phosphorus in soil. In order to test these hypotheses, a growing season field experiment was conducted to determine soil total phosphorus, available phosphorus, inorganic phosphorus fractions, soil bacterial community composition and diversity, and phosphorus transformation key gene abundance by adding cow dung and its biochar to the soil planted with *P.euphratica* seedlings.

## 2 Materials and methods

### 2.1 Study area

The study area was located in a forest garden nursery (44° 01 ′ N, 87° 65 ′ E) in Midong District, Urumqi City, Xinjiang Uygur Autonomous Region, China. The tested soil was gray desert soil. The fundamental characteristics of the soil included a bulk density of 1.25 g/cm^3^, a pH of 8.02, a CEC of 20.32 cmol/kg, an SOM of 22.15 g/kg, a total N content of 1.31 g/kg, a total P content of 0.92 g/kg, a total K content of 21.4 g/kg, an AN of 65.15 mg/kg, an Olsen-P content of 17.26 mg/kg, and an AK content of 351.54 mg/kg.

### 2.2 Biochar materials

Biochar produced from raw cow dung was collected from beef cattle farmers in the Midong District, Urumqi City, Xinjiang Uygur Autonomous Region. Its properties were as follows: pH 7.9, Total N 4.39 g/kg, Total P 3.94 g/kg, specific surface area 3.12 m^2^/g, pore size 2.11 nm, ash 17.18%, C (%) 25.15, H (%) 6.47, O (%) 29.99, N (%) 1.85. After drying the collected cow dung for a week, it was burned at 500°C for 2 h under anaerobic conditions (Henan Lize Environmental Protection Technology Co., Ltd.), let stand for 2 h, and finally cooled to room temperature. A 2 mm sieve was used to completely mix the produced biochar. Its properties were as follows: pH 10.38, Total N 11.87 g/kg, Total P 10.96 g/kg, specific surface area 7.58 m^2^/g, pore size 3.82 nm, ash 25.37%, C (%) 40.52, H (%) 4.58, O (%) 9.03, N (%) 4.23. Cow dung biochar was chosen because cow dung is a cheap, high-quality renewable resource that can be commercially obtained in large quantities, in addition to which it may be applied to enhance utilization efficiency and lower environmental pollution caused by the improper utilization of cow dung resources.

### 2.3 Experimental design

In this study, three treatments were tested: the control (CK); the cow manure biochar returning treatment (BC); and cow dung returning treatment (CD). The amount of cow manure biochar returning to the field was 2.63 t hm^-2^ (35% carbon yield from 7.5 t hm^-2^ cow manure pyrolysis carbonization); and the amount of cow dung returning to the field was 7.5 t hm^-2^. The plot size was 7.5 m×10 m, row spacing was 50 cm, distance between plots was 1 m, and protective rows were placed all around. A randomized block design was used, and each treatment was performed in triplicate. In late March, 2-year-old *P.euphratica* seedlings with similar plant heights, and intact healthy roots were selected for planting. Cow dung and its biochar were spread on the soil surface prior to planting the seedlings and thoroughly mixed into the soil using a rotary tiller. Plants were not stressed by drought; therefore, only regular field irrigation and management was necessary. At the end of the growth phase in late October, *P.euphratica* seedlings were collected. The stems and roots of these seedlings were separated, and biomass (kg plant^-1^) was measured. Next, the seedlings were subjected to an oven temperature of 105°C for 30 min and then dried at 80°C for 48 h until their weights remained constant. Plant samples were ground into powder and digested with concentrated sulfuric acid, following which phosphorus concentrations in the leaves, stems, and roots were determined via colorimetry. Soil samples including rhizosphere (RS) and non-rhizosphere (NRS) areas were extracted from each treatment. Loose soil, which was first shaken off, and soil still adhering to roots, which was brushed off, were collected to form a composite rhizosphere sample from each plot. After removing plant residues and stones, they were divided into two parts with a 2 mm screen and air-dried for soil characteristic analysis, following which the fresh part was stored at − 80°C for soil DNA extraction.

### 2.4 Soil basic physical and chemical properties

Soil pH was determined using a pH meter (Leici, Shanghai, China) with a soil-to-deionized water ratio of 12.5 (w/v). Combustion infrared absorption spectroscopy was used to determine the total carbon content. Organic matter was determined using a potassium dichromate-sulfuric acid solution. The ammonium acetate technique was used to assess cation exchange capacity (pH = 7). Kjeldahl and flame photometric methods were used to calculate total nitrogen and total potassium. Alkali-hydrolyzed nitrogen and available potassium were determined using the alkaline hydrolysis diffusion method and the neutral ammonium acetate solution extraction-flame photometer method, respectively. Inductively coupled plasma mass spectrometry (ICP-MS) was used to measure the total amounts of Al and Fe.

### 2.5 Determination of soil phosphorus characteristics

Phosphomolybdate colorimetry was used to calculate the total phosphorus content of soil. The amount of available P in the soil was measured using the molybdenum-antimony colorimetric technique and sodium bicarbonate extraction. The approach developed was used to classify phosphorus (inorganic phosphorus fraction).

### 2.6 DNA extraction, amplification, sequencing and sequence data processing

The chloroform fumigation extraction method was used to extract soil microbial biomass phosphorus, and the soil microbial content was measured using ammonium molybdate-ascorbic acid colorimetry.

Beijing Baimaike Biotechnology Co., Ltd. performed the high-throughput sequencing of 16S rDNA PCR products. A TGuide S96 Magnetic Soil/Stool DNA Kit (Tiangen Biochemical Technology Co., Ltd, Beijing, China) was used to extract soil DNA, according to the manufacturer’s instructions. The quality and quantity of extracted DNA were determined using 1.8% agarose gel electrophoresis, while the concentration and purity of DNA were determined using a Nanodrop ND-2000 (Thermo Scientific, Wilmington, USA) ultraviolet photometer. Amplification of the hypervariable region V3-V4 of the bacterial *16S rRNA* gene was performed using the primers 338F(5’-ACTCCTACGGGAGGCAGCA-3’) and 806R(5’-GGACTACHVGGGTWTCTAAT-3’)(Aleklett et al., 2015). The overall volume of the PCR reaction was 10μL, which included a 5–50 ng DNA template, 0.3μL of downstream and upstream primer (10μM each), 5μL of KOD buffer, 2μL of 2 mmol L^-1^ dNTPs, and 0.2 μL of KOD polymerase; PCR amplification protocol steps were, pre-denaturation at 95°C for 5 min, 20 cycles of denaturation at 95°C for 30 s, annealing at 50°C for 30 s extension at 72°C for 40 s, and final extension at 72°C for 7 min. An Omega DNA purification kit (Thermo Scientific, Wilmington, USA) was used to purify the amplified products, and Qseq-400 (Houze Biotechnology Co., LTD, Hangzhou, China) was used to quantify them. Primer sequences were located and eliminated using Cut Adapt. USEARCH (v10) was used to obtain the PE reads, and UCHIME was used to eliminate chimeras. The above-mentioned steps produced high-quality reads, which were then used for the analysis. USEARCH (v10) clustered sequences showing a similarity > 97% into the same operational taxonomic unit (OTU) and filtered out OTUs appearing less than twice in all samples. The SILVA classification library (http://www.arb-silva.de) was used to categorize sequences.

### 2.7 Quantification of functional genes

Fluorescence quantitative PCR (qPCR) was performed by Shenzhen Weikemeng Technology Group Co. Ltd. The manufacturer’s recommendations were strictly followed when using the FastDNA SPIN kit (MP Biomedicals, California, USA) to harvest soil DNA. A Nanodrop ND-2000 (Thermo Scientific, Wilmington, USA)was used to assess DNA content and quality. The primers used in the experiments are listed. DNA extraction involved the repetition of three gene amplification techniques and was performed in a real-time fluorescence quantitative MA-6000 (Yarui Biotechnology Co., LTD, Suzhou, China) using an AceQ ® qPCR SYBR ® Green Master Mix kit(Vazyme, Nanjin, China). The reaction system was a combination of SYBR and primers (0.4 μL of 10 μM PCR-specific primer F and 0.4 μL of 10 μM PCR-specific primer R). The system consisted of 8 μL mixture A and 8 μL template dilution sample. The temperature settings for the real-time PCR reaction were 95°C for 5 min, 95°C for 15 s, 60°C for 30 s, and 52°C for annealing. Melting curve analysis was performed to confirm the specificity of the reaction. A plasmid standard was constructed, and a standard curve was drawn. The standard R-values for *phoC, phoD, gcd, and pqqC* were 0.9986, 0.9974, 0.9975, and 0.998.

### 2.8 Data analysis

The data were subjected to statistical analysis using the SPSS software (version 23.0; SPSS Inc., Chicago, IL, USA). One-way analysis of variance (ANOVA) followed by the LSD test was used, with statistical significance being set at *P* < 0.05. Based on the Spearman correlation matrix, the pheatmap package and R package vegan were used for cluster analysis. RDA was performed using the vegan package. One-way analysis of variance (ANOVA) was performed in R, and Shapiro-Wilk normality test was used for data (logarithmic transformation method was used for non-normal data). The data of variance homogeneity test were adopted by Bartlett test of homogeneity of variances. P < 0.05 was used as the significance threshold to compare the data differences, and the agricolae package was used for calculation.

## 3 Results

### 3.1 Effects of cow dung returning methods on rhizosphere and non-rhizosphere soil properties and growth of *P.euphratica* seedlings

The cow manure biochar returning treatment caused a significant increase in the Ph of both non-rhizosphere (0.19) and rhizosphere soils (0.15), compared to the control group ((*P* < 0.05). By contrast, direct application of cow dung returning treatment caused a significant reduction in the pH of both soil types (0.31 and 0.33, respectively); (*P* < 0.05). Addition of cow dung and biochar significantly increased CEC, SOM, Total C, and Total N in both rhizosphere and non-rhizosphere soils compared to those of the control group (*P* < 0.05). The CEC, SOM, Total C, and Total N contents in non-rhizosphere soil showed significant increases of 36%, 17%, 79%, and 38% (*P* < 0.05), respectively, whereas those in rhizosphere soil exhibited significant increases of 40%, 4%, 107%, and 40% (*P* < 0.05), respectively. Comparing the two methods of returning cow dung to the field indicated that the increase in CEC, SOM, Total C and Total N content in rhizosphere and non-rhizosphere soil by the biochar returning treatment was greater than that of the direct cow dung returning treatment. Compared with those of the control group, utilization of biochar led to a significant increase in the levels of Total K, Total Al, and Total Fe in both rhizosphere and non-rhizosphere soils, whereas introducing cow dung directly resulted in a significant decrease in the amount of Total Al in both rhizosphere and non-rhizosphere soils. The AN and AK contents in non-rhizosphere and rhizosphere soils modified with cow dung and cow manure-biochar were increased significantly (*P* < 0.05). Considering the two methods of returning cow dung to the field, the increase in total K, total Al, total Fe, AN, and AK content in rhizosphere and non-rhizosphere soil caused by the cow manure-biochar returning treatment was greater than that of the direct cow dung returning to the field treatment.

Both returning methods significantly increased the biomass of roots, stems, and leaves of *P.euphratica* seedlings, compared with the control group (**Table 1**). However, when comparing the two return methods, the increase in plant biomass was not significant. Both methods significantly increased the phosphorus content of roots, stems, and leaves of *P.euphratica* seedlings (*P* < 0.05) compared with the control. Cow manure biochar returning showed a more significant increase in the phosphorus content of the roots, stems, and leaves than the other two methods (*P* < 0.05).

**Table 1:** Effect of cow dung return and cow dung biochar return on biomass (g dry weight plant-1) and phosphorus content (g kg-1) of poplar seedlings.

	CK	BC	CD
Root biomass(g plant^-1^)	124.05 ± 11.26 b	135.45 ± 10.56 a	134.26 ± 12.62 a
Stem biomass(g plant^-1^)	284.62 ± 23.85 b	300.59 ± 19.26 a	298.49 ± 20.47 a
Leaf biomass(g plant^-1^)	78.39 ± 7.66 b	95.15 ± 10.94 a	92.46 ± 13.52 a
Total biomass(g plant^-1^)	487.06 ± 38.45 c	531.19 ± 39.49 a	525.21 ± 36.15 b
Root P concentration(g kg^-1^)	0.73 ± 0.12 c	1.22 ± 0.14 a	1.05 ± 0.10 b
Stem P concentration(g kg^-1^)	1.18 ± 0.27 c	1.54 ± 0.30 a	1.39 ± 0.25 b
Leaf P concentration(g kg^-1^)	1.92 ± 0.35 c	2.35 ± 0.28 a	2.18 ± 0.25 b
Total P concentration(g kg^-1^)	3.83 ± 0.68 c	5.11 ± 0.59 a	4.62 ± 0.51 b

All values are mean ± standard error (n = 3); CK indicates no biochar added; BC indicates cattle manure added; CD indicates cattle manure added; different letters in the same row indicate statistically significant differences between treatments (*P* < 0.05), same letters in the same row indicate non-statistically significant differences between treatments (*P* < 0.05).

### 3.2 Effects of cow dung returning methods on phosphorus availability and morphology in rhizosphere and non-rhizosphere soil of *P.euphratica* seedlings

**Figure 1:**
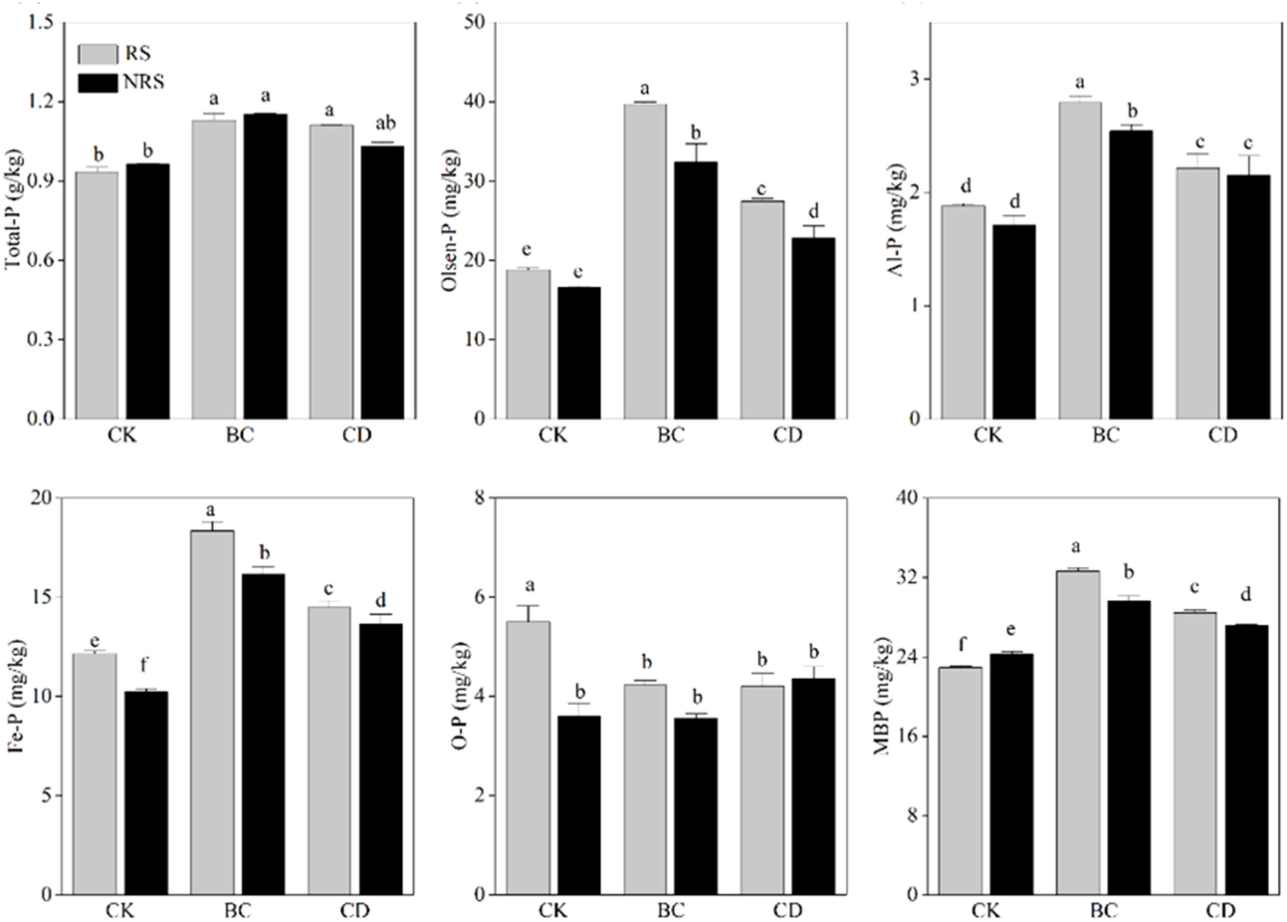
Effect of cow dung return and cow dung biochar return on Total P, Olsen-P, Al-P, Fe-P, O-P and MBP content of inter- and non-inter-rooted soils. Error bars indicate standard error of the mean (n=3). BC indicates cow dung biochar added; CD indicates cow dung added; NRS non-inter-rooted; RS inter-rooted. Different letters between treatments indicate significant differences (*P* < 0.05).

The contents of Total-P, Olsen-P, Al-P, Fe-P, O-P, and MBP in rhizosphere and non-rhizosphere soils of direct cow dung returning and cow-manure biochar returning are shown (**Figure 1**). Compared with that in the control, direct cow dung returning to the field significantly increased the total-P content in rhizosphere and non-rhizosphere soils by 22.1% and 21.8%, respectively, while cow manure biochar returning to the field significantly increased total-P in rhizosphere and non-rhizosphere soils by 22.4% and 22.9%, respectively. Compared with the direct cow dung returning, cow manure biochar returning to the field did not significantly increase Total-P in rhizosphere and non-rhizosphere soils. **(Figure 1A)**. Compared with the control, direct cow dung returning to the field resulted in 75% and 46% increases in the Olsen-P content in rhizosphere soils and non-rhizosphere soils, respectively. However, cow manure-biochar returning significantly increased the Olsen-P content in rhizosphere and non-rhizosphere soils by 134% and 110%, respectively. Thus, the treatment of cow manure-biochar returning to the field caused a more significant increase in the Olsen-P content in both rhizosphere and non-rhizosphere soils (**Figure 1B**). Compared to the control, the cow dung returning and cow manure biochar returning significantly increased the contents of Al-P and Fe-P in rhizosphere and non-rhizosphere soils, with the cow manure biochar returning treatment improving Al-P and Fe-P in rhizosphere and non-rhizosphere soils more significantly (**Figsures 1C, D**). The difference between changes in the O-P content in rhizosphere and non-rhizosphere soils did not show significant variability in regard to the two methods of returning (**Figure 1E**). In comparison to the control group, direct cow dung returning led to 24% and 11% increases in MBP in rhizosphere and non-rhizosphere soils, respectively. However, cow manure biochar returning resulted in a more significant increase in MBP, with 42% and 22% increases in rhizosphere and non-rhizosphere soils, respectively. The increase in MBP was most significant when cow manure biochar was used to return cow dung (**Figure 1F**).

### 3.3 Composition and diversity of soil bacterial community

**Figure 2:**
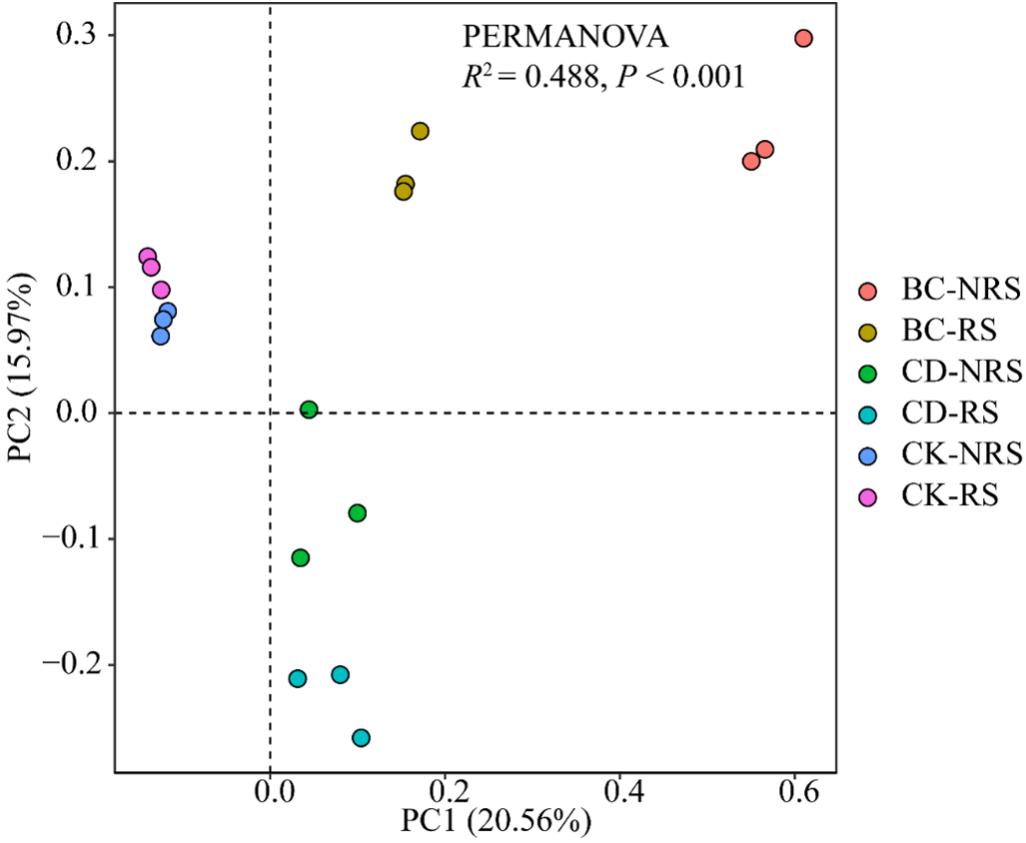
PCoA ranking of the structure of the inter-root and non-inter-root soil bacterial communities for different cow manure return methods and control treatments. BC indicates cow dung biochar added; CD indicates cow dung added; NRS non-inter-rooted; RS inter-rooted.

Diversity analysis requirements were met when sequencing depth was sufficient, as indicated by the Coverage index of 100% (**Table 2**) displaying the microbial diversity index. In each treatment, the Shannon and Chao1 indices exhibited significant differences (*P* < 0.05), with the BC treatment showing significantly higher values than the CD and CK treatments. The Shannon diversity indices of the BC treatment were 1.11 times and 1.10 times that of CD and CK treatments, respectively, while those of the Chao1 diversity index were 1.20 times and 1.15 times that of CD and CK treatments, respectively. The PCOA ordination diagram did not indicate significant differences between CD and CK treatments. The first principal component (PC1) showed a contribution rate of 20.56%, while the second principal component (PC2) showed a contribution rate of 15.97% (**Figure 2**). The findings indicated a clear pattern in the distribution of microbial communities among the six treatment groups with a significant distance between them, implying considerable variation in microbial communities across the groups. The multivariate permutation variance test indicated that there was a significant difference (*P* < 0.001) between microbial community structures. Differences between the microbial community structures corresponding to BC, CD, and CK were particularly evident, and the points of the three groups were distributed in different quadrants. On PC1 (X-axis), the two cow dung return methods were significantly different from the control, whereas the cow dung direct return treatment was close to the control sample. It was also distinguished along PC2 (y-axis) under the two cow dung return methods. In summary, the soil bacterial community structure is affected differently by various methods of cow dung application.

**Table 2:** Bacterial community diversity index based on 16S rRNA genes (minimum sequence similarity of 90%).

Soil sample	Coverage	Shannon	Chao1
BC-NRS	100 ± 0 a	6.68 ± 0.27 a	783.00 ± 27.81 a
BC-RS	100 ± 0 a	6.84 ± 0.06 a	788.67 ± 32.41 a
CD-NRS	100 ± 0 a	6.03 ± 0.05 b	639.33 ± 22.67 b
CD-RS	100 ± 0 a	6.12 ± 0.06 b	672.00 ± 35.76 b
CK-NRS	100 ± 0 a	6.11 ± 0.04 b	673.67 ± 24.88 b
CK-RS	100 ± 0 a	6.13 ± 0.02 b	693.00 ± 16.01 b

CK indicates no addition; BC indicates addition of cow dung biochar; CD indicates addition of cow dung; different letters in the same column indicate significant differences between treatments (*P* < 0.05).

**Figure 3:**
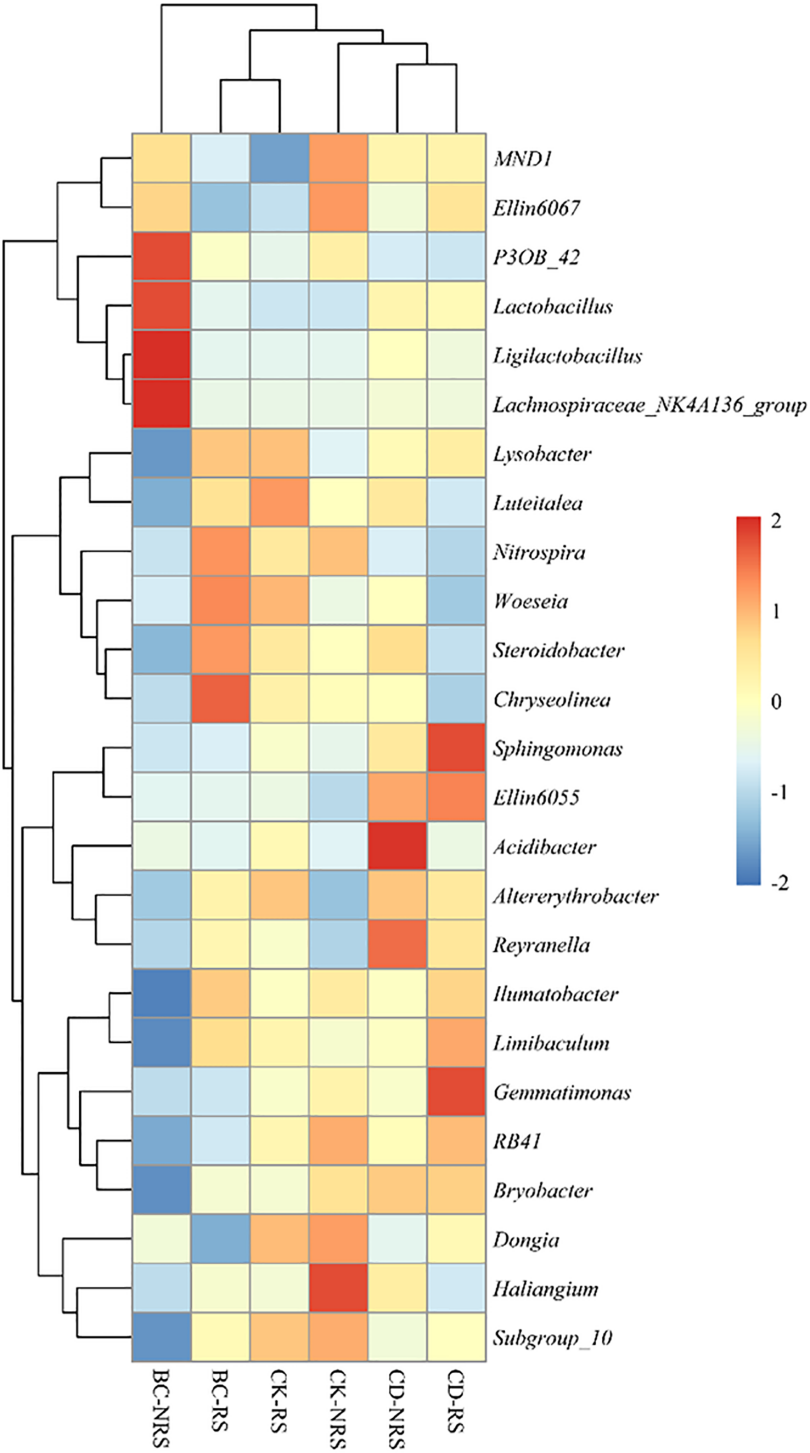
Stratified clustering and heat map of bacterial abundance in the first 25 OTUs of inter- and non-inter-rooted soil for different cattle manure return methods and control treatments. Each column in the heat map represents a sample and each row represents a taxonomic level. The colour scale indicates the abundance of genetic species, expressed as the standard deviation of the mean, with red indicating high abundance and blue indicating low abundance. BC indicates cow dung biochar added; CD indicates cow dung added; NRS non-inter-rooted; RS inter-rooted.

The relative abundances of dominant microbial genera (top25) in each treatment are shown, indicating that the top 25 genera accounted for 22–28% of the total abundance (**Figure 3**). The BC-NRS treatment group showed significant increases of 1.4%, 1.1%, 7.52%, and 2.27% in the relative abundances of *P3OB_42, Lactobacillus, Ligilactobacillus, and Lachnospiraceae_NK4A136*_group, respectively, compared to the other five groups. In BC-RS treatment, the abundances of *Nitrospira, Steroidobacter, and Chryseolinea* were significantly higher, being 1.68%, 1.19%, and 1.49%. In the CK-RS treatment, the abundances of *Lysobacter* and *Woeseia* were significantly higher, being 2.06% and 0.47%. In the CK-NRS treatment, the *Haliangium* group exhibited a significantly higher relative abundance of 1.59% compared to other groups. In the CD-RS treatment, the relative abundances of *Sphingomonas, Ellin6055, and Gemmatimonas* were significantly greater compared to those of the other treatments (4.33%, 0.79%, and 0.98%, respectively).

**Figure 4:**
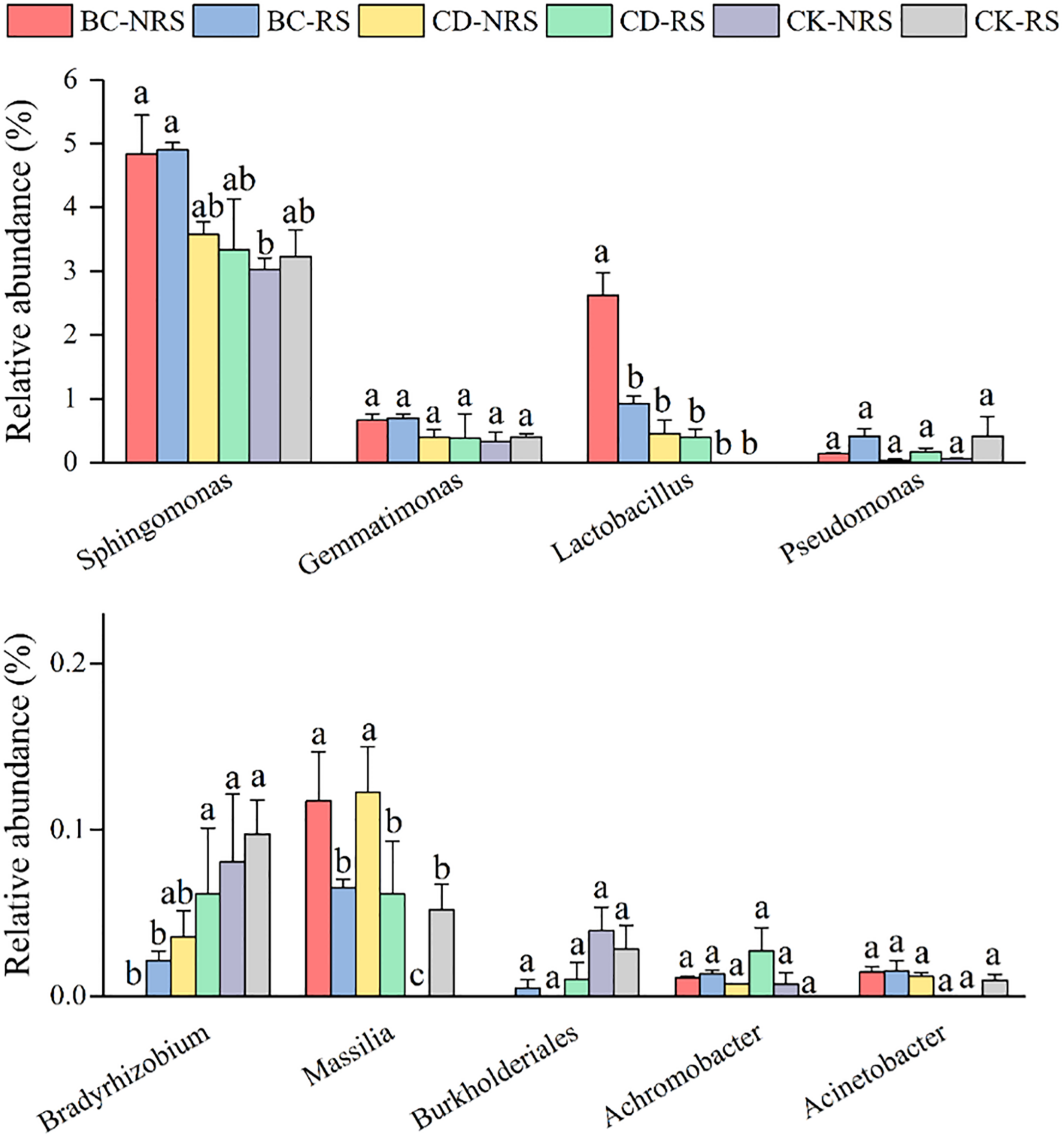
Comparison of the relative abundance (mean ± SE, n = 3) of inter- and non-inter-root soil phosphorus dissolving bacteria for different cow dung return methods and control treatments. BC indicates cow dung biochar added; CD indicates cow dung added; NRS non-inter-rooted; RS inter-rooted. Different letters between treatments indicate significant differences (*P* < 0.05).

The relative abundances of bacteria capable of solubilizing phosphate are shown in **Figure 4**. The abundances *of Sphingomonas, Gemmatimonas, Lactobacillus*, and *Pseudomonas* were significantly higher, with average relative abundances of 3.82%, 0.48%, 0.73%, and 0.20%, respectively. The relative abundances of *Sphingomonas* and *Lactobacillus* in the BC-NRS treatment group were significantly higher compared to those of the other groups, with respective relative abundances of 4.83% and 2.62%. the CK-NRS and CK-RS treatments had relative abundances of 0.07% and 0.09%, respectively.

### 3.4 Factors affecting bacterial community

**Figure 5:**
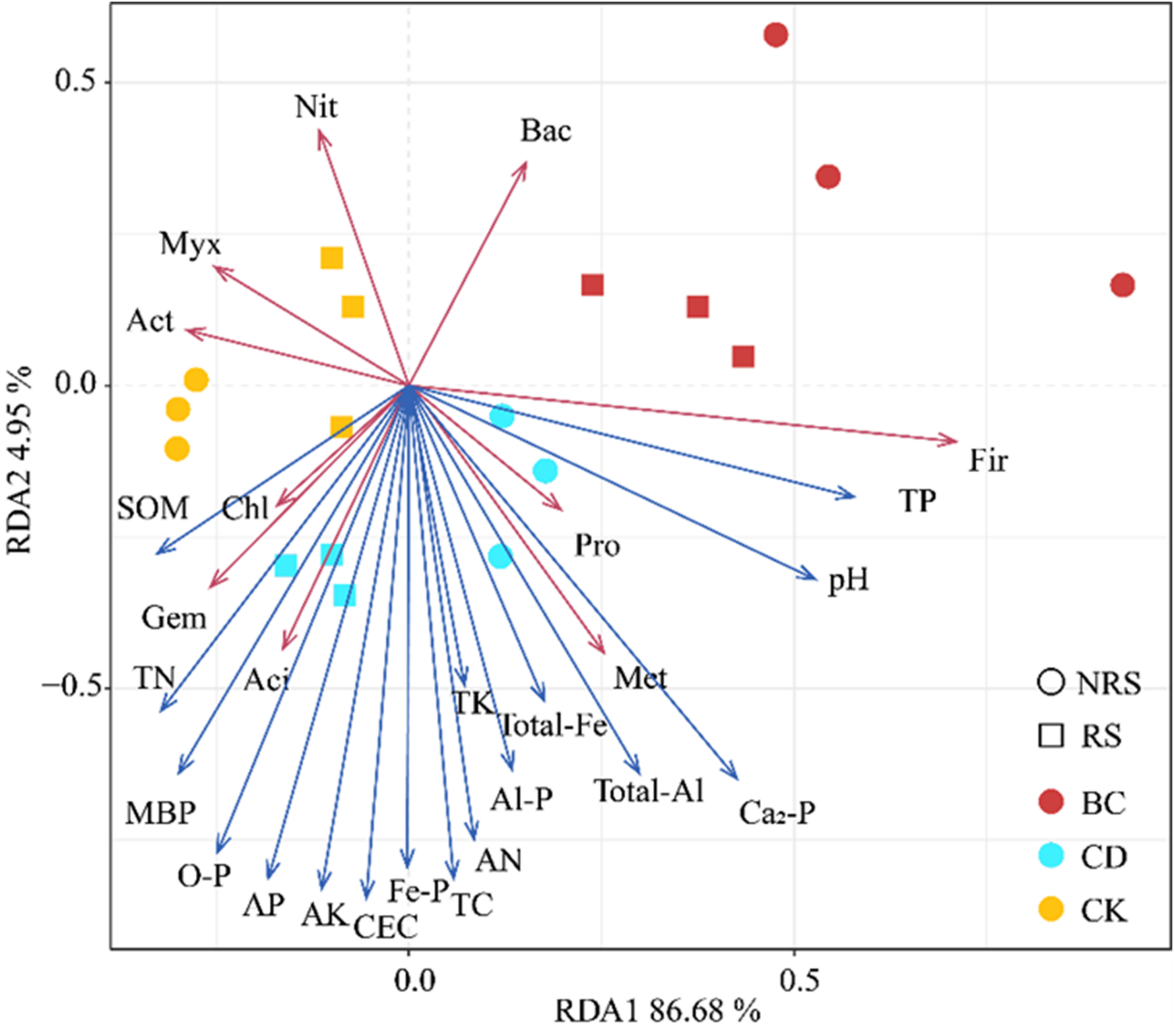
Redundancy analysis (RDA) of inter- and non-inter-root soil bacteria and soil physicochemical properties for different cattle manure return methods and control treatments. CK indicates no addition; BC indicates addition of cow dung biochar; CD indicates addition of cow dung.

Redundancy analysis was used to analyze the effects exerted by soil properties on the abundance of bacterial phyla in rhizosphere and non-rhizosphere soils of *P.euphratica* seedlings. The association between soil properties and the abundance of bacteria is shown in **Figure 5**. Notably, 17 soil properties explained 91.63% of the variance in the abundance of bacteria in rhizosphere and non-rhizosphere soils of *P.euphratica* seedlings. Among these TC, CEC, AN, MBP, AK, AP, Fe-P, and O-P exerted the greatest effect on the abundance of bacteria in rhizosphere and non-rhizosphere soils of *P.euphratica* seedlings, followed by TN, total-Al, Ca2-P, and Al-P, which exerted the least effect. TP was positively correlated with Fir; Pro was positively correlated with pH; Met was positively correlated with total Al, total Fe, and Ca_2_-P; and Aci was positively correlated with AP, O-P, and MBP. Furthermore, Gem was positively correlated with TN, while Chl was positively correlated with SOM.

### 3.5 Effects of cow dung returning methods on the abundance of phosphorus functional genes in rhizosphere and non-rhizosphere soil of *P.euphratica* seedlings

**Figure 6:**
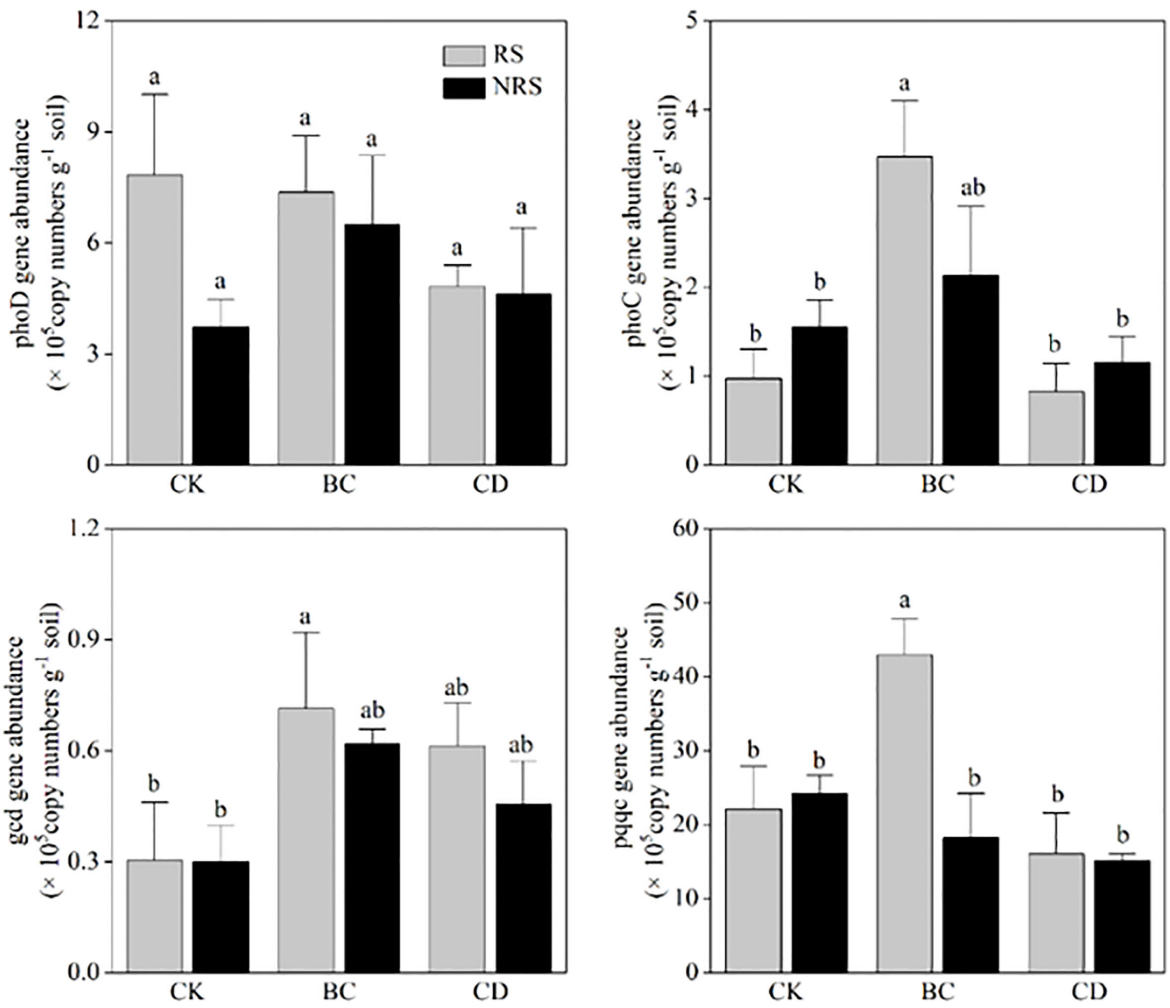
Quantitative analysis of phosphorus mineralization and solubilization genes in inter-root and non-root soils of different cow dung return methods and control treatments (copies g^-1^). Error bars indicate the standard error of the mean (n=3). BC indicates cow dung biochar added; CD indicates cow dung added; NRS non-inter-rooted; RS inter-rooted. Different letters between treatments indicate significant differences (*P* < 0.05).

The effects of returning cow dung on the abundance of phosphorus functional genes in rhizosphere and non-rhizosphere soils of *P.euphratica* seedlings are shown (**Figure 6**). Compared with the control group, the effects of returning cow dung and biochar on *phoD* copy number in rhizosphere and non-rhizosphere soils were not significant, and there was no significant difference between the effects exerted by the two return methods on *phoD* copy numbers. Compared with that of the control group, the effect exerted by cow dung return on the copy number of *phoC* gene was not obvious, whereas biochar return significantly increased the copy number of *phoC* in rhizosphere and non-rhizosphere soil (*P* < 0.05). Compared with those of the two return methods, the effect of biochar return on the copy number of *phoC* was more significant. Compared with the control group, both methods increased the copy number of *gcd* in rhizosphere and non-rhizosphere soils; however, there was no significant difference between the copy numbers of *gcd* corresponding to the two methods. Biochar return significantly increased the copy number of *pqqc* in rhizosphere soil (*P* < 0.05), whereas none of the other treatments exerted a significant effect.

## 4 Discussion

### 4.1 Comparison of soil chemical properties between cow dung returning and carbonization returning

The results indicated that, compared with the control, both cow dung returning and cow manure biochar returning changed the chemical properties of soil, and cow manure biochar returning exerted a greater effect on soil properties than direct return. Compared with the direct cow dung returning, cow manure biochar returning increased the soil pH, CEC, SOM, Total C, Total N, Total K, Total Al, Total Fe, AN, and AK contents. Hence, cow dung application methods appeared to have exerted varying degrees of effects on the chemical characteristics of soil. The pH of biochar was influenced by the rate of carbonization, pyrolysis temperature, and type of raw material (Weber and Quicker, 2018). Pyrolysis of cow dung at 500°C yielded biochar products that had a higher pH compared to original cow dung, consequently elevating the pH level of soil. Compared with cow dung, cow dung biochar produced various functional groups during pyrolysis, such as hydroxyl and carboxyl groups, which adsorbed highly oxidized organic matter on the biochar, thereby increasing soil CEC (Tomczyk et al., 2020). The use of high-temperature pyrolysis in biochar production will lead to a decrease in the abundance of acidic functional groups, especially carboxyl functional groups, while introducing more alkaline functional groups. Biochar produced at high temperatures shows a highly aromatic composition and a well-structured carbon layer (Aon et al., 2023). Our results demonstrated that biochar would increase SOM and Total C in soil (**Table 3**) and improve soil quality by increasing microbial activity and carbon sequestration in soils. The improvement in soil nutrients caused by cow manure biochar may be attributed to two factors. The cow dung itself has high organic matter and nutrient content. During pyrolysis, nutrients are decomposed at high temperatures, causing organic nutrients in the biomass to be converted into inorganic forms available to plants (Qian et al., 2019). As a result, the inorganic nutrient concentration in cow dung biochar was greater than that in cow dung. Biochar has the characteristics of hydrophilicity, high internal porosity, high surface area and relatively polar surface chemistry, which is due to the formation of oxygen-containing functional groups. Therefore, when biochar is incorporated into the soil, it absorbs the declining gravity water and reduces the permeability of water, so that soil moisture and nutrients can be more easily preserved in the soil, which has a positive impact on the nutritional status of the soil. (Nyambo et al., 2023).

**Table 3:** Effect of cow dung return and cow dung biochar return on soil properties in different root zones.

Treatment	pH	CEC (cmol/kg)	SOM (g/kg)	Total C (%)	Total N (g/kg)	Total K (g/kg)	Total Al (g/kg)	Total Fe (g/kg)	AN (mg/kg)	AK (mg/kg)
CK-NRS	7.97 ± 0.01 c	21.34 ± 0.11 e	23.44 ± 0.16 d	2.25 ± 0.01 d	1.34 ± 0.02 e	21.42 ± 0.01 d	64.21 ± 0.03 c	33.66 ± 0.06 b	70.39 ± 1.55 d	353.04 ± 1.75 e
CK-RS	8.02 ± 0.01 b	21.73 ± 0.13 e	23.25 ± 0.2 d	2.16 ± 0.02 e	1.22 ± 0.02 f	20.92 ± 0.03 f	63.25 ± 0.1 d	31.96 ± 0.14 d	64.66 ± 2.5 e	339.25 ± 2.4 f
BC-NRS	8.16 ± 0 a	29.06 ± 0.04 b	27.33 ± 0.09 a	4.03 ± 0.02 b	1.85 ± 0.01 a	21.64 ± 0.03 c	68.71 ± 0.14 a	35.12 ± 0.03 a	95.17 ± 1.34 a	441.74 ± 1.69 b
BC-RS	8.17 ± 0.01 a	30.42 ± 0.15 a	24.17 ± 0.05 c	4.48 ± 0.05 a	1.71 ± 0.02 c	22.11 ± 0.02 a	67.01 ± 0.05 b	33.86 ± 0.17 b	91.75 ± 2.16 b	485.53 ± 3.34 a
CD-NRS	7.66 ± 0.01 e	24.13 ± 0.08 d	24.27 ± 0.05 c	2.51 ± 0.01 c	1.82 ± 0.01 b	21.28 ± 0.04 e	63.37 ± 0.07 d	34.6 ± 0.2 a	90.28 ± 1.06 c	410.71 ± 2.32 d
CD-RS	7.69 ± 0.01 d	24.87 ± 0.06 c	25.83 ± 0.11 b	2.5 ± 0.01 c	1.58 ± 0.01 d	21.96 ± 0.05 b	61.92 ± 0.39 e	32.76 ± 0.33 c	81.91 ± 2.16 d	436.29 ± 5.44 c

All values are mean ± standard error (n = 3); different letters in the same column indicate statistically significant differences between treatments according to the least significant difference (LSD) test (*P* < 0.05). Total C, Total N, Total K, Total Al, Total Fe, AN, and AK.

### 4.2 Comparison of soil phosphorus availability between cow dung returning and carbonized returning

The results indicated that, compared with the control, both cow dung returning and cow manure biochar returning changed the availability of phosphorus in soil, and that cow manure biochar returning exerted a greater impact on the availability of phosphorus in soil than direct cow dung returning. Compared with the direct returning of cow dung, the Olsen-P and MBP contents in the rhizosphere and non-rhizosphere soils were increased by the former(Hinsinger, 2001). Thus, various methods of returning cow dung may exert dissimilar effects on phosphorus availability in soil. This observation may be attributed to two possible factors. Biochar derived from biomass (especially livestock manure and urban sewage) is rich in nutrients, so it can directly increase the level of available phosphorus in soil (Shi et al., 2023). The phosphorus in cow dung, such as in phospholipids and phytic acid, exists mainly in organic form, making it difficult for crops to absorb and utilize it. During high-temperature pyrolysis, the O-P bond is broken, converting organic phosphorus into inorganic phosphorus (Uchimiya and Hiradate, 2014), releasing phosphate, and directly increasing the available phosphorus content in the soil. This is consistent with the results of (Jin et al., 2023; Duan et al., 2022), they found that phosphorus in biochar itself can directly increase the content of available phosphorus in soil. Therefore, whereas cow dung mainly adds organic phosphorus to the soil, biochar produced after carbonization treatment may directly provide soil-available phosphorus (El-Naggar et al., 2019; Xu G. et al., 2014; Zhai et al., 2015). Due to the different metal components in the raw materials, Al 3 + and Fe 3 + were released after pyrolysis at high temperature and combined with soluble phosphorus to form metal-phosphorus complexes such as Al-P and Fe-P, which increased the content of Al-P and Fe-P in the soil (Hei et al., 2023).Compared with directly returning cow dung, the effect of returning cow manure biochar on total-P content was not significant, probably because the biochar produced from a similar amount of cow dung was added to the soil, without adding an extra phosphorus source. The second reason may be that the method of returning cow dung to the field changed the chemical properties of soil, thereby indirectly affecting the availability as well as the form of phosphorus in alkaline soil (Wang et al., 2021). Various crucial factors, including pH as well as Fe^3+^ and Al^3+^, may influence the availability and speciation of phosphorus in alkaline soils, as demonstrated by previous studies (Arif et al., 2017; Wei et al., 2021). The levels of Fe-P in the soil were significantly elevated compared to those of the control group, as indicated by their contents (**Table 3**). Although high-temperature thermal interpretation of raw materials released AL^3+^ and Fe^3+^, which combined with soluble phosphorus to form metal-phosphorus complexes, such as Al-P and Fe-P, thereby increasing phosphorus fixation, this is offset by the fact that biochar can adsorb ions in the soil that can easily react with phosphorus and precipitate cations, such as Fe^3+^ and AL^3+^. Alternatively, the formation of chelates between Fe3+ and AL3+ and organic molecules adsorbed on the surface of the biochar may release trapped phosphorus, thereby enhancing the availability of soil phosphorus (Xu et al., 2014). Studies have demonstrated that biochar enhances CEC, elevates pH, and liberates organic ligands. These alterations boost the absorption of cations, such as Fe3+ and AL3+, and weaken the retention and sedimentation of phosphorus, ultimately resulting in a surge of available phosphorus in the soil (Cui et al., 2011; DeLuca et al., 2015b; Nahidan and Ghasemzadeh, 2022).

### 4.3 Effects of biochar on microbial community

The results of this study indicated that introducing cow manure biochar results in a greater diversity of bacterial communities in both the rhizosphere and non-rhizosphere soils of *P.euphratica* seedlings. This result is consistent with those of previous studies. After one year of cow manure biochar application, the diversity of soil bacteria significantly increased, and the relative abundance of nitrifying bacteria increased (Nguyen et al., 2018). According to Zhang et al. (2019), *Proteobacteria* in red soil are notably affected by both biochar and fertilizers. Zhang et al. (2017) found that biochar treatment increased the abundance and diversity of bacterial communities in the tobacco rhizosphere, compared to the control. Biochar application has been shown to alter soil properties, ultimately affecting soil microorganisms and altering the composition of soil bacterial communities. Because biochar contains a large amount of soluble nutrient elements, it can directly affect the physical and chemical properties of soil and has a positive effect on the activity of soil microorganisms (Ameloot et al., 2014). In this study, pH was positively correlated with *Pro*, TN was positively correlated with *Gem*, and SOM was positively correlated with *Chl*. Under the conditions of this experiment, we speculate that the influence of microbial abundance may be affected by the significant increase of pH, TN and SOM (Wu et al., 2023; Yuan et al., 2023). Previous studies have shown that biochar may directly or indirectly affect soil bacteria. Firstly, they may directly increase the number of soil bacteria by providing food sources for microorganisms, and secondly, biochar itself has a rich pore structure and large specific surface area, thereby indirectly providing habitats and shelter for microorganisms, increasing the water-holding capacity of soil, and improving the living environment of soil bacteria. The addition of cow dung biochar promoted the growth and reproduction of bacteria in the rhizosphere and non-rhizosphere soils of *P.euphratica* seedlings, thereby changing the soil bacterial community structure.

### 4.4 Effects of biochar on phosphate-solubilizing bacteria and phosphorus transformation functional genes

Phosphate-solubilizing bacteria, constituting 1–50% of the total number of microorganisms, act as a pivotal biological factor that enhances the availability of phosphorus in soil. Biochar can indirectly affect the availability of phosphorus in soil by changing the abundance of phosphate-solubilizing bacteria. Reportedly, phosphate-solubilizing bacteria are capable of dissolving insoluble phosphorus in soil by secreting organic acids, which directly dissolve phosphate minerals via anion exchange reactions with PO_4_^3-^ or chelation reactions with cations, such as Fe^3+^ and AL^3+^. In this study, we found that soil treated with cow manure biochar exhibited a significant increase in the abundance of certain phosphate-solubilizing bacteria, which finding is substantiated by prior research findings. According to Zheng et al. (2019)), the abundance of inorganic phosphate-solubilizing bacteria is augmented by straw biochar. Biochar increases the activity of phosphate-solubilizing bacteria in the soil and provides an eco-friendly niche for their survival. Liu et al. (2017) reported that incorporating rice husk biochar produced at 400°C into the soil enhanced soil quality and promoted the growth of phosphate-solubilizing bacteria, resulting in improved soil phosphorus effectiveness. Thus, the application of biochar supplements the soil with a large amount of phosphorus, thereby improving the availability of phosphorus in the soil, which in turn facilitates the growth and development of plants by providing sufficient phosphorus and weakening the ability of phosphate-solubilizing bacteria to dissolve phosphorus. Moreover, the presence of biochar did not affect or diminish the population of phosphate-solubilizing bacteria; therefore, abiotic factors may play a dominant role in the impact of biochar on phosphorus availability.

## 5 Conclusion

The findings of the current study showed that returning cow dung in different forms may exert varying effects on soil properties, seedling growth, soil phosphorus availability, microbial communities, and microbial functional genes. The method of returning biochar to the field is more effective in increasing the available phosphorus (Olesn-P) content than the method of directly applying cow dung. Biochar improved phosphorus availability in rhizosphere and non-rhizosphere soils, both directly and indirectly, by inducing changes in the chemical and microbial properties of soil. Of the different return methods, biochar return affected soil microbial communities and changed the composition and diversity of bacterial communities (e.g., increased bacterial biodiversity) in rhizosphere and non-rhizosphere soils. In summary, carbonizing cow dung resources (e.g., cow manure biochar) and returning them to the soil shows potential as a sustainable method that can be applied to effectively improve soil phosphorus availability, as well as soil phosphorus status and crop phosphorus health. At the same time, reducing the large amount of investment in land phosphorus fertilizer and using organic fertilizers and their biochar for partial replacement can also activate soil and crop rhizosphere microbial activity and promote the growth of phosphorus-related microorganisms. It is suggested that the use of biochar should be increased in the management of plantation, and the long-term combined analysis of biochar utilization methods of Populus euphratica seedlings and cow dung should be carried out in order to find the most scientific addition method.

## Data availability statement

The original contributions presented in the study are included in the article/**Supplementary Material**. Further inquiries can be directed to the corresponding author.

## Author contributions

Conceptualization, YF. Methodology, YDC and YLC. Software, YDC. Validation, ZL. Formal Analysis, GL. Investigation and Project Administration. All authors contributed to the article and approved the submitted version.
