# Supplementary material for: Differential effects of cow dung and its biochar on Populus euphratica soil phosphorus effectiveness, bacterial community diversity and functional genes for phosphorus conversion
Source: Front Plant Sci. 2023 Sep 14;14:1242469. doi: 10.3389/fpls.2023.1242469 (PMC10538999; doi:10.3389/fpls.2023.1242469)
Supplement: Supplementary file 1 [file Table_1.docx]

Supplementary data, Table 1

| Sample ID | Raw Reads | Clean Reads | Denoised Reads | Merged Reads | Non-chimeric Reads |
| --- | --- | --- | --- | --- | --- |
| BC-NRS1 | 80,330 | 80,022 | 76,429 | 68,649 | 60,147 |
| BC-NRS2 | 80,009 | 79,685 | 74,799 | 62,192 | 51,876 |
| BC-NRS3 | 80,055 | 79,709 | 77,229 | 66,488 | 48,176 |
| BC-RS1 | 41,175 | 41,023 | 38,472 | 34,261 | 30,333 |
| BC-RS2 | 59,902 | 59,646 | 56,832 | 51,156 | 45,322 |
| BC-RS3 | 59,992 | 59,745 | 56,869 | 50,951 | 44,758 |
| CD-NRS1 | 80,026 | 79,606 | 75,731 | 67,406 | 57,414 |
| CD-NRS2 | 80,108 | 79,767 | 76,283 | 68,040 | 57,600 |
| CD-NRS3 | 79,920 | 79,548 | 75,997 | 69,432 | 59,915 |
| CD-RS1 | 79,994 | 79,687 | 74,739 | 60,068 | 46,442 |
| CD-RS2 | 80,109 | 79,706 | 76,080 | 69,241 | 60,117 |
| CD-RS3 | 79,956 | 79,601 | 75,996 | 68,033 | 59,061 |
| CK-NRS1 | 80,149 | 79,797 | 76,171 | 68,802 | 59,109 |
| CK-NRS2 | 79,795 | 79,442 | 75,261 | 64,583 | 53,605 |
| CK-NRS3 | 79,897 | 79,521 | 75,765 | 68,035 | 58,949 |
| CK-RS1 | 76,840 | 76,500 | 73,155 | 67,022 | 58,708 |
| CK-RS2 | 80,148 | 79,821 | 75,911 | 68,443 | 60,106 |
| CK-RS3 | 72,239 | 71,941 | 67,676 | 56,995 | 46,325 |
