# Supplementary material for: Differential effects of cow dung and its biochar on Populus euphratica soil phosphorus effectiveness, bacterial community diversity and functional genes for phosphorus conversion
Source: Front Plant Sci. 2023 Sep 14;14:1242469. doi: 10.3389/fpls.2023.1242469 (PMC10538999; doi:10.3389/fpls.2023.1242469)
Supplement: Supplementary file 2 [file Table_2.docx]

Supplementary data, Table 2

| Data normality test and variance homogeneity test | | | | | | |
| --- | --- | --- | --- | --- | --- | --- |
| index | units | Shapiro-Wilk normality test | | Bartlett test of homogeneity of variances | | Data distribution |
| TC | % | W = 0.75594 | p-value = 0.1038 | Bartlett's K-squared = 8.0202 | p-value = 0.1551 | Logarithmic transformation |
| TN | g/kg | W = 0.94179 | p-value = 0.3108 | Bartlett's K-squared = 1.9972 | p-value = 0.8495 | Normal distribution |
| Total Al | g/kg | W = 0.88407 | p-value = 0.2673 | Bartlett's K-squared = 13.345 | p-value = 0.0767 | Logarithmic transformation |
| Total Fe | g/kg | W = 0.93393 | p-value = 0.2276 | Bartlett's K-squared = 8.0979 | p-value = 0.1509 | Normal distribution |
| TK | g/kg | W = 0.93796 | p-value = 0.2673 | Bartlett's K-squared = 3.5752 | p-value = 0.612 | Normal distribution |
| TP | g/kg | W = 0.918 | p-value = 0.2746 | Bartlett's K-squared = 11.665 | p-value = 0.1141 | Logarithmic transformation |
| pH | —— | W = 0.82557 | p-value = 0.1052 | Bartlett's K-squared = 13.121 | p-value = 0.0742 | Logarithmic transformation |
| CEC | cmol/kg | W = 0.85938 | p-value = 0.1324 | Bartlett's K-squared = 3.0947 | p-value = 0.8423 | Logarithmic transformation |
| Ca2-P | mg/kg | W = 0.88843 | p-value = 0.0713 | Bartlett's K-squared = 11.124 | p-value = 0.1256 | Logarithmic transformation |
| Ca8-P | mg/kg | W = 0.95739 | p-value = 0.5522 | Bartlett's K-squared = 9.731 | p-value = 0.08322 | Normal distribution |
| Al-P | mg/kg | W = 0.8717 | p-value = 0.0687 | Bartlett's K-squared = 7.7782 | p-value = 0.2623 | Logarithmic transformation |
| Fe-P | mg/kg | W = 0.94011 | p-value = 0.2911 | Bartlett's K-squared = 3.8933 | p-value = 0.5649 | Normal distribution |
| O-P | mg/kg | W = 0.9157 | p-value = 0.1085 | Bartlett's K-squared = 3.7846 | p-value = 0.5808 | Normal distribution |
| Ca10-P | mg/kg | W = 0.78328 | p-value = 0.1322 | Bartlett's K-squared = 8.4201 | p-value = 0.4136 | Logarithmic transformation |
| AN | mg/kg | W = 0.91468 | p-value = 0.104 | Bartlett's K-squared = 1.6664 | p-value = 0.8931 | Normal distribution |
| SOM | g/kg | W = 0.87174 | p-value = 0.0921 | Bartlett's K-squared = 5.0887 | p-value = 0.1219 | Logarithmic transformation |
| AK | mg/kg | W = 0.82794 | p-value = 0.4416 | Bartlett's K-squared = 3.6659 | p-value = 0.3212 | Logarithmic transformation |
| AP | mg/kg | W = 0.91223 | p-value = 0.0613 | Bartlett's K-squared = 16.747 | p-value = 0.0659 | Logarithmic transformation |
| MBP | mg/kg | W = 0.94479 | p-value = 0.3493 | Bartlett's K-squared = 3.9027 | p-value = 0.5635 | Normal distribution |

Note：A Shapiro-Wilk normality test greater than 0.05 indicates a normal distribution; Bartlett test of homogeneity of variances greater than 0.05 means homogeneity of variance
